# Six Weeks of Supplementation with Bovine Colostrum Effectively Reduces URTIs Symptoms Frequency and Gravity for Up to 20 Weeks in Pre-School Children

**DOI:** 10.3390/nu15163626

**Published:** 2023-08-18

**Authors:** Maciej Hałasa, Karolina Skonieczna-Żydecka, Bogusław Machaliński, Leonard Bühner, Magdalena Baśkiewicz-Hałasa

**Affiliations:** 1 Department of General Pathology, Pomeranian Medical University in Szczecin, Powstańców Wlkp. 72, 70-111 Szczecin, Polandleonard.buehner@gmx.net (L.B.); poziomka@pum.edu.pl (M.B.-H.); 2 Department of Biochemical Science, Pomeranian Medical University in Szczecin, Broniewskiego 24, 71-460 Szczecin, Poland

**Keywords:** bovine colostrum, upper respiratory tract infections, pre-school children

## Abstract

Bovine colostrum is considered to provide anti-infective protection. Here, we present the first randomized controlled trial (RCT) aimed at assessing the preventive use of colostrum against upper respiratory tract infections (URTIs) in healthy pre-school children. We analyzed 57 children—35 in the colostrum (COL—dried bovine colostrum) and 22 in the placebo (PBO—dried whey) group, who received these substances as follows: first 15 days 2 × 500 mg and then 30 days 1 × 500 mg. The reporting on the children’s health status, specifically on the frequency and gravity of URTI symptoms and abdominal side effects, was performed via an online survey. The influence of colostrum on the frequency of days with URTI symptoms remained significant until the 20th week of observation and reached 31% of median reduction. The median reduction reached 37% when the gravity of symptoms was analyzed. When we grouped symptomatic days into episodes of second gravity level, the reduction in their frequency was even larger (50%) and lasted until the end of the trial (21 weeks). No significant side effects, especially abdominal, were reported during the trial. Colostrum supplementation in pre-school children is well tolerated, safe and provides protection from frequency of URTIs and their gravity.

## 1. Introduction

Of all childhood health issues, upper respiratory tract infections (URTIs) seem to be the most common problems, according to various authors, as they typically occur 3–8 times a year [1,2,3,4]. Paradoxically, they are also probably the most underrated type of pediatric diseases. The reason is that although their symptoms are predominantly mild, they lead to the loss of many school days and workdays. Thus, they are usually perceived as obstacles in organizing daily activities for parents and guardians, rather than the real medical issues they constitute for their children. Moreover, the economic burden of these infections is frequently brought to attention [5]. This common view on URTIs should raise some concerns, since the occurrence of frequent childhood infections, especially those where antibiotics were used, has been found to correlate with health problems at a later age [3,5,6,7].

Limiting the number and gravity of URTIs with the use of various approaches has been a goal of medicine throughout its entire history, usually with a limited degree of success. Traditionally, the preventive attempts included the use of several natural products derived from plants or animals, including probiotic supplementation [8,9,10]. With the development of more advanced laboratory methods, there has also been a rise in popularity of extracted or synthesized vitamins, minerals and other elements considered as necessary supplements for health preservation [10,11,12,13,14]. Some of these supposedly health-promoting materials, often combined into multicomponent dietary supplements, produced promising results in clinical tests, though doubts about their effectiveness still remain valid [10,12,13,14,15,16]. A similar lack of efficacy can be observed when attempting to reduce the extent of already developed infections with the use of currently available treatment methods [1,17]. The exceptions are bacterial infections, for which, according to some authors, antibiotics can potentially reduce the URTI’s duration [3,14,18,19]. However, the absolute majority of uncomplicated URTIs, especially in the childhood, is of viral origin, and the antiviral medication is either still unavailable for many pathogens or too expensive to be used in less severe infections [1,20]. The intervention types offered by modern pharmacology in most cases of URTIs are typically limited to symptomatic or accessory treatments reducing pain, fever and other symptoms, such as nasal congestion or cough [3]. Most of these interventions are regarded as not safe for young/pre-school children [21]. Consequently, patients have to rely on their own defenses, predominantly the development of the child’s adaptive immunity, specifically towards particular pathogens. Unfortunately, until now, there have been no commercially available vaccines specifically for upper respiratory tract viruses other than influenza [1].

Bovine colostrum has long been claimed to have the potential to modulate and boost human immunity to infections. However, valid clinical tests have only proven this for selected groups of subjects, such as those represented by either extreme physically active or heavily stressed out and overworked adult individuals [22,23,24]. Although the trials on the use of colostrum against recurrent URTIs in children brought rather enthusiastic results, they have so far been limited to practically two observational studies only, with no control groups treated with a placebo [22,23]. This lack of randomized controlled trials (RCTs) most probably results from the ethical concern of performing pediatric trials, where the control group participants have to be deprived of the access to the tested product. While this dilemma may be valid when the potentially effective medication or method is tested, it is not in the case of a supplement, which does not belong to the primarily recommended range of medical interventions. In addition to testing the populations with recurrent URTIs, there have been two other studies performed with either allergic children suffering from the recurrent respiratory infections, receiving complex supplement containing bovine colostrum among other components, [25] or immunodeficient subjects receiving colostrum [26]. Both of these RCTs have brought the results in favor of the colostrum-treated groups.

Considering the above and recognizing the great importance of conclusively establishing whether the bovine colostrum is capable of preventing URTIs in healthy children, we decided to perform a triple-blind placebo-controlled trial in an attempt to resolve this issue. The trial was performed concomitantly with a group of pre-school children attending one of the only two kindergarten facilities participating in the study. This was aimed to reduce the bias resulting from participants being exposed to unpredictable pathogens’ variety related to seasonal shift and to various environmental factors. Our team recently conducted a survey-based study on the effectiveness of colostrum in prevention of URTIs among medical university students [27]. In this study, we followed the same environmental and seasonal strategies as before. Data collection in this trial was performed with an online-based questionnaire that was enabled by the EU Survey tool. The experience we gained from the study with university students allowed us to effectively organize a similar experiment with the pre-school children. Our experiment is the first placebo-controlled trial to date that tests the efficacy of bovine colostrum in preventing URTIs in children.

## 2. Materials and Methods

### 2.1. Recruitment, Blinding and Randomization

All trial subjects were pre-school children (3–7 years of age; mean, 4.3 ± 1.21 years; male to female ratio—1:0.8) attending one of the two large (over 300 pupils) public kindergartens facilities in Szczecin, Poland. Trial participants were invited through the advertisements posted in these facilities. The only exclusion criterion was an allergy to cow’s milk. The general health status of all participants was considered to be good, suitable for participating in kindergarten activities.

Trial administration, which included the enrolment process, collection of formal consent forms, blinding and randomization of participants and database maintenance, was performed by a dedicated person—the administrator. He was also the only person who contacted participants via email and provided them with links to the online survey forms (both the initial and daily/weekly surveys). The polls the participants responded to were based on the EU Survey system, an official online survey management tool provided for free by the European Commission [28].

After receiving the approval by the Ethical Committee of the Pomeranian Medical University in Szczecin, the study also obtained the acceptance from the managements of both facilities where the children were enrolled. The recruitment of participants began with a series of meetings where parents and guardians were provided with a comprehensive explanation of the study principles in both verbal and written form. They were given the opportunity to ask any questions regarding their child’s participation in the trial.

We obtained formal consent forms from the parents and guardians of 89 participants. Upon recruitment, the administrator immediately assigned a trial ID number to each participant, by which they were referred to for the rest of the trial. This way, the identity of all children remained anonymous to all investigators (including cooperating kindergartens managements), as well as other participants (parents and guardians), until the completion of data analysis. Upon blinding, the random assignment to tested groups was performed with the MS Excel Random Sort tool. Out of 89 participants recruited, we were able to form the colostrum (COL) group consisting of 45 children and the placebo (PBO) group consisting of 44 children.

### 2.2. Outline of the Trial and Participation Issues

**Figure 1:**
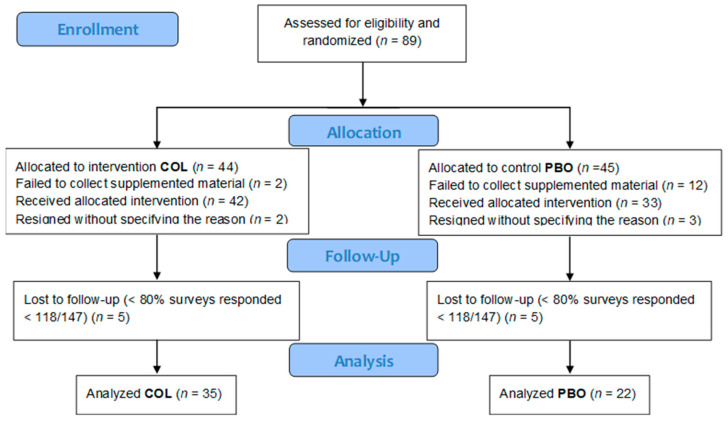
The CONSORT flow diagram.

To ensure a more coherent population, particularly in terms of exposure to infectious agents responsible for the URTIs, we included children from just two kindergartens in the trial. This helped to minimize potential variations in exposure and create a more homogenous study population. To increase the consistency of trial conditions further, we conducted the study in exactly the same timeframe between the end of September and mid-February, when the risk of contracting the URTIs is highest in our geographic zone. The rationale behind such a design was fully explained in the report from our previous study with medical university students [27]. The design of the trial is presented in the CONSORT diagram (Figure 1).

After blinding and randomization processes were completed by 29 September 2022, the parents and guardians were invited to collect the supplemented material sufficient for the entire period of the trial. The appropriate packages identifiable only by the trial ID numbers were distributed by the management of the kindergartens in which the trial was conducted. At this stage, 14 participants (2 from the COL group and 12 from the PBO group) were excluded because of not collecting the supplement despite the repeated summons. We found no rational explanation for such a disparity in the collection of supplements between the COL and the PBO group participants.

Immediately after the beginning of supplementation, 3 additional participants resigned (1 from the COL group and 2 from the PBO group) without specifying any reason. After 4 months of the trial, 2 additional participants resigned (1 from the COL and 1 from the PBO groups), again without providing the reason for withdrawal. Out of the 70 participants who remained in the trial until its completion, we made the decision to exclude an additional 13 participants (5 from the COL group and 8 from the PBO group) from the final analysis, due to their poor consistency in responding to daily surveys. The minimal response rate was established at 80% of daily surveys.

The trial started on 1 November 2022, upon the signal given by the administrator to all participating children. Over the first 15 days, the participants were taking 2 of the 500 mg sachets of supplementation material per day. They were supposed to take one sachet in the morning and one in the evening on an empty stomach, with water or with a teaspoon of natural yogurt. Then, for the following 30 days, the administration schedule consisted of just one sachet in the morning, and after 45 days, the supplementation period terminated.

In addition to that, the parents and guardians were instructed to give 8 consecutive doses of the supplementation material (twice daily for 4 days) to their children whenever symptoms of newly developing URTI occurred until the end of the trial. The trial lasted for 147 days until 24 February 2023, when daily surveys were terminated.

### 2.3. Supplementation Material

The tested materials’ packages, consisting of 100 unlabeled pouches containing either COL or PBO, were provided free of charge by Genactiv Trade Sp. z o. o., Poznań, Poland. The COL sachets contained a mixture of 500 mg of freeze-dried bovine colostrum obtained within 2 h after calf delivery, mixed with 500 mg of dried banana powder. The PBO doses consisted of 500 mg of spray-dried whey mixed with 500 mg of dried-out banana. These were the typical supplementation materials’ doses that were efficiently used in our previous experiments, causing measurable biological effects [27,29,30]. Allocation concealment was assured by using sequentially numbered drug containers of identical appearance containing identical, unlabeled sachets, while the addition of the banana powder gave both the tested material and the placebo an identical taste and smell.

### 2.4. Surveys

The online surveys allowed us to gather all the information on the health status and other important health-related parameters from trial participants. These surveys were provided to their parents and guardians through direct links sent via email, using the EU Survey tool [28]. These survey results were collected by the administrator individually from each participant. Each survey questionnaire remained available for responding or editing over a period of 7 days from publication date.

Three types of surveys were used in this trial were (1) initial, (2) daily and (3) weekly. Through the initial survey, we obtained the data needed for descriptive statistics and an overview of the characteristics of the trial participants. The daily surveys were the most important source of data for our experiment. Their questionnaire consisted of 6 questions, with the most important of them focusing on the participants’ health status, particularly inquiring about the severity of the URTI symptoms. Our weekly surveys served to enumerate days with any potential gastrointestinal side effects.

The inquiry about the gravity of the URTIs symptoms was based on the following original scale, effectively introduced in our previous study, [27] further called the URTI score: 0 points—no symptoms; 1 point—mild symptoms not influencing regular daily activity (sporadic sneezing and/or cough, mild sore throat, runny/blocked nose of low intensity—at least one of these symptoms with no fever); 2 points—mediocre symptoms reducing regular daily activity (cough, runny/blocked nose, pain in area of sinuses—at least one of these symptoms with body temperature < 38.0 °C); 3 points—severe symptoms prohibiting regular daily activity (1st group of symptoms: persisting, intense cough, runny/blocked nose of significant intensity, 2nd group of symptoms: serious headache, muscle ache, loss of appetite, serious fatigue, thermic discomfort—combination of at least one from the 1st group and two from the 2nd group of symptoms together with fever of ≥38.0 °C). Based on the numerical scales created in this manner, we were able to grade the intensity of subjective parameters, such as the severity of self-observed symptoms.

### 2.5. Outcomes

Our first primary outcome was the frequency of days with any URTI symptoms (FSD—frequency of symptomatic days). This was the ratio calculated based on enumerating days with presence of any degree of URTI symptoms (1–3 URTI score) and dividing this by the number of days within the period of interest (Table 1).

Our second primary outcome was the average gravity score (AGS) of URTI symptoms. This was a ratio calculated after summarizing the URTI scores (0–3) and dividing them by the number of days within the period of interest.

Both of the primary outcomes were analyzed over the entire 21-week (147 days) period of the experiment, as well as within several periods, namely 4, 8, 12, 14, 16, 18 and 20 weeks counted from the beginning of supplementation.

Our secondary outcome was the number of URTI episodes (NUE) over the entire trial period. This required finding a subjective definition of an episode, which would fit the most accurate medical description of URTI and allow for a credible enumeration of such episodes in our trial. We decided to define an URTI episode as at least 3 days of symptoms at the 2nd severity level, being separated by at least 3 asymptomatic days.

### 2.6. Statistical Analysis

Statistical analyses were performed with the use of Med Calc statistical software version 22.110 (Ostend, Belgium). Two-sided *p* < 0.05 indicated a statistically significant difference. The normality of continuous variables distribution was verified using the Shapiro–Wilk test. Consequently, continuous data were expressed as median ± interquartile range (IQR) and compared using the Mann–Whitney test. Categorical data were expressed as numbers (percentages) and compared using chi-square or Fisher’s exact test. The significance level for the type I error was set at 0.05.

### 2.7. Bioethical Approval

The trial was performed in accordance with the protocol approved by the Pomeranian Medical University Bioethics Committee (KB-006/24/2022) on 18 May 2022.

## 3. Results

### 3.1. Study Group Characteristics

We analyzed data from 57 children—35 in the COL group and 22 in the PBO group. There were no significant differences regarding gender distribution between groups (*p* = 0.15). The baseline characteristics of analyzed participants are shown in Table 2.

### 3.2. Colostrum Decreases Frequency of Days with URTI Symptoms

**Figure 2:**
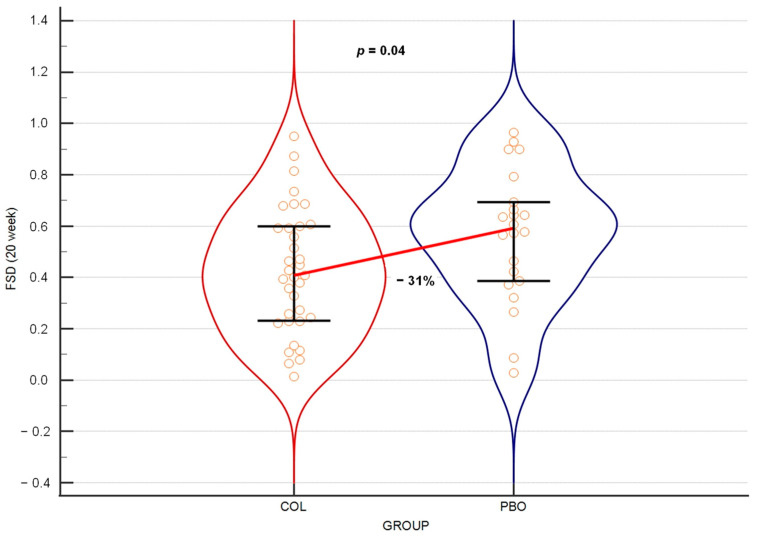
Violin plot on the frequency of days with any symptoms of URTI over 20 weeks of the trial. The horizontal line connects medians. Orange circles depict individual results. Error bars represent interquartile ranges.

The main measure of URTIs’ occurrence used in our experiment was the frequency of days with URTI symptoms (FSD) over various periods of the trial. When analyzing all days with any severity level of URTI symptoms over a period of 21 weeks from the beginning of the trial supplementation, which was the entire period of daily survey collection, the results were found to be at the border of statistical significance (*p* = 0.052), with a 26% reduction in URTI frequency in the COL group compared to the PBO group. When the same parameter was tested after 20 weeks of the trial, it produced a statistically significant result (Figure 2).

Testing the same at weeks 8, 12, 16 and 18 of the trial produced statistically significant differences between the COL and the PBO groups, with the reduction in FSD medians ranging between 30% and 37% (Table 3).

### 3.3. Colostrum Reduces Average Gravity Scores of URTI Symptoms

**Figure 3:**
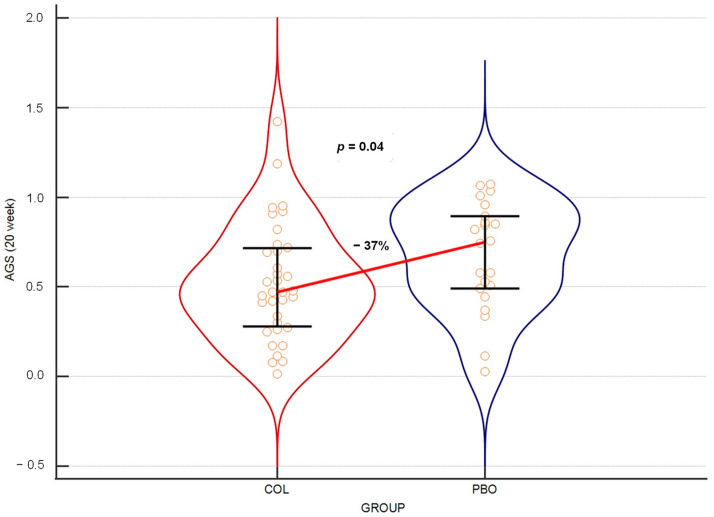
Violin plot of the medians of URTI symptoms’ average gravity scores over the 20 weeks of the trial. The horizontal line connects medians. Orange circles depict individual results. Error bars represent interquartile ranges.

Our second primary outcome, the average gravity score of URTI symptoms (AGS), was assessed based on reporting to daily surveys on the intensity of symptoms according to the grading scale described in the Material and Method section. Parallel to the result obtained with FSD, when this parameter was analyzed over 21 weeks, the results reached a near-significant level (*p* = 0.052), with a 36% reduction in the URTI score median in the COL group compared to the PBO group. However, when this was tested over 20 weeks, the results turned out to be statistically significant (Figure 3).

At weeks 4, 8, 12, 14, 16 and 18 of the trial, testing the same parameter showed that colostrum provided variable but consistently statistically significant protection from the gravity of URTIs, as assessed by the reduction in AGS medians in the COL group compared to the PBO group. This reduction ranged between 33% and 45% (Table 4).

### 3.4. Colostrum Reduces Number of URTI Episodes

**Figure 4:**
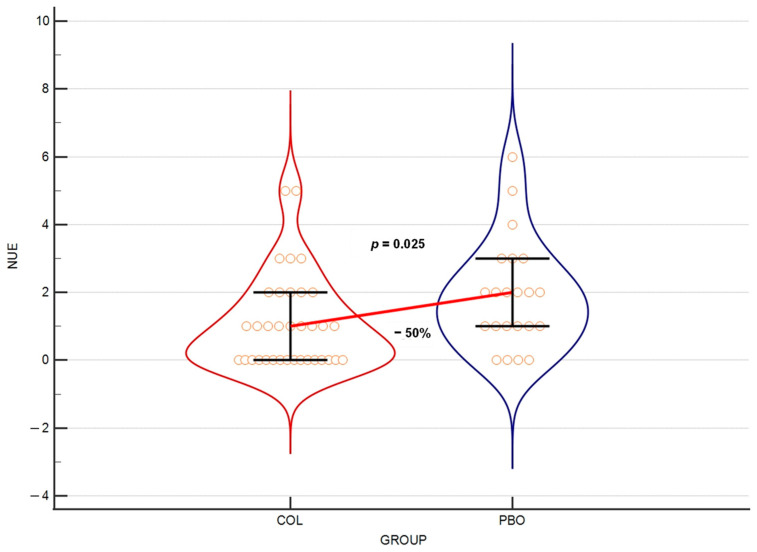
Violin plot of the number of URTI episodes scored with gravity 2 level over the entire study (21 weeks). The horizontal line connects medians. Orange circles depict individual results. Error bars present interquartile ranges.

Due to the difficulty in defining URTI events, especially based on the self-reported URTI symptom days, we decided to use the number of URTI episodes (NUE) over the entire duration of the trial as a secondary outcome. At week 21 of the trial, there was a statistically important difference between the result obtained in the COL group versus the PBO group (*p* = 0.025), with a 50% reduction in these events’ median in the COL group (Figure 4).

### 3.5. Colostrum Produced No Gastrointestinal Side Effects

**Figure 5:**
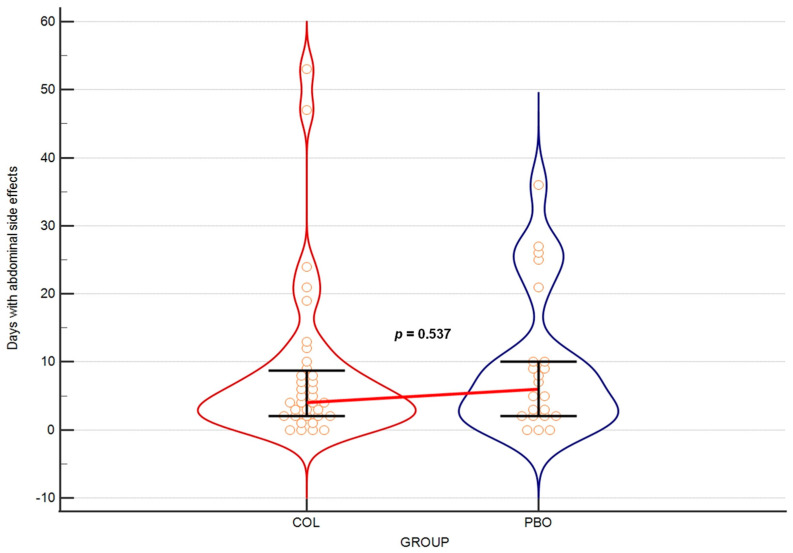
Days with abdominal side effects over the entire study. The horizontal line connects medians. Orange circles depict individual results. Error bars present interquartile ranges.

Over the entire course of the trial (21 weeks), we observed no serious side effects that would lead to a discontinuation of supplementation. The reports on the symptoms (bloating, abdominal pain and diarrhea) involving the gastrointestinal tract showed no difference between the COL and the PBO groups (*p* = 0.537) (Figure 5).

## 4. Discussion

The evidence leading to a justified conclusion that bovine colostrum provides effective prevention against the URTIs is building up. Our results demonstrating this effect in terms of frequency and severity of URTIs in pre-school children come from the first-ever reported randomized, triple-blind, placebo-controlled trial performed with a general, presumably healthy population. Previous reports from the pediatric field, predominantly presenting very promising data on the effectiveness of colostrum in the prevention of respiratory, as well as gastrointestinal infections, were based on observational cohort studies [24,31,32] or studied children with co-existing health problems, such as allergies or immunodeficiencies [21,26]. Most of the already reported RCTs have restricted testing the colostrum efficacy in prevention of URTIs to physically active adult (mainly athletes) populations [22,23,24]. The only RCT results showing the effectiveness of colostrum against URTIs in young adults of a nonathletic background, specifically the population of medical university students, have just recently been reported by our team [27]. Another important remark is that our current experiment, unlike most of the other trials, but similar to the abovementioned study, was based on main supplementation given to participants only for the first 45 days, while the observation lasted for 147 days. In fact, the proportion of supplementation to observation periods in our trial was the smallest of all RCTs testing colostrum use against URTIs.

### 4.1. Colostrum Reduces URTI Frequency and Severity

Our two main measures of colostrum effectiveness in the prevention of URTIs were the frequency of symptomatic days (FSDs) and the URTI symptoms’ average gravity score (AGS). The information on URTI symptoms’ appearance was self-reported by parents and guardians based on a gravity scale named the URTI score (grades 0–3; see Materials and Methods).

For the first primary outcome, the FSD, we summed up all days reported with any symptoms (no matter the gravity level) and used the sum to calculate the frequency of days with URTIs within particular periods of the experiment. By the 20th week of the trial, we observed a statistically significant reduction in the FSD median by a third in the COL as compared to the PBO group. The analyses of the periods between the beginning of supplementation and weeks 4, 8, 12, 14, 16, 18, 20 and 21 revealed that the colostrum supplementation started to exert its significant protective impact on the appearance of days with URTI symptoms no earlier than by the week 8. This protective effect was then observed at week 12, as well as weeks 16, 18 and 20, while at weeks 14 and 21, the results were borderline significant (*p* = 0.0501 and *p* = 0.052, respectively).

Our second primary outcome was the AGS of URTI symptoms. The statistically significant reduction in AGS medians was observed in the COL vs. the PBO group when the period from beginning of supplementation until week 20 was assessed. The repeated measures of periods ending at weeks 4, 8, 12, 14, 16 and 18 also consistently showed a significant result in this regard, while the borderline significance was found at week 21 (*p* = 0.052).

When AGS medians’ reduction induced by colostrum supplementation is confronted with FSD results, a more consistent statistically significant difference in favor of the COL group over all analyzed periods is observed. Especially striking is the difference observed in AGS in the period ending at week 4, while there is no such difference for FSD, thus suggesting the earlier development of a protective effect against the gravity versus frequency of URTIs. The outcome medians’ reduction rate also favors the gravity reduction, ranging between 33% and 45% for AGS, while for FSD, this range is just from 30% to 37%. While it has not been confirmed by any test, the above-presented reasoning allows us to assume that even if URTIs develop in colostrum-protected children, they seem to have a milder course. However, more studies are needed to verify this notion.

### 4.2. URTI Episodes

We also made an attempt to assess the number of URTI episodes over the entire trial period. However, this parameter was found to be of limited value, mainly due to the necessity of manipulating the raw data in order to manually enumerate the episodes, as well as the challenge of accurately defining URTI episodes. For the purpose of this analysis, we defined the most probable episodes as being those of the second severity level (mediocre symptoms) and lasting for at least 3 days, with 3 days of separation from another event. The difficulty with this approach was that many symptomatic days could not be included in the identifiable episodes. In some instances, they were not part of a ≥3-day-long sequence or, in other cases, the separation of such sequences was not long enough (<3 days). The latter case was the main problem when we initially attempted to count episodes of all severity levels, including those with the mildest symptoms. The symptoms of the first gravity level were sometimes reported over long periods without a 3-day separation between them, thus making it impossible to accurately calculate the individual URTI episodes of all severity levels.

Another problem with enumerating the URTI episodes is that the old definition of the common-cold event seems to be currently less practical. The classical description of such an event was that it typically lasted for about 7 days and appeared with mild-to-moderate symptoms [33]. Since this definition was first given, a large variety of approaches have become available to ease the URTI symptoms, which may largely change the presentation of the disease. Although many of these treatments are not advised to be used in the young children, the decision in this regard was left to parents and guardians, and such supportive, mainly symptomatic treatments were not forbidden in the trial [21]. Consequently, the inconsistent course of diseases in our trial may depend on participants receiving such treatments. This fact may partially decrease the value of the analyses based on the comparison of episodes between the COL and the PBO groups (Figure 4). As a result, despite obtaining a strong, statistically significant 50% reduction in the median number of second-gravity-level URTI episodes in the COL group vs. the PBO group over the entire 21-week trial period, we consider this parameter to be a secondary outcome of the study.

### 4.3. How Colostrum Use May Prevent the URTIs

Many experiments, of both in vitro and in vivo types, have demonstrated various influences of colostrum or its components on individual immune system elements, which may translate into a boosting of the anti-infectious resistance [34,35]. Additionally, reports from clinical trials, assessing the overall protective effect of colostrum supplementation in infections, have been published, confirming the same effect [23,24,25,26,36,37]. However, to present the most comprehensive picture of how colostrum may positively influence the immunity against URTIs, a brief pathophysiology review is required.

The absolute majority of URTIs, even exceeding 90% of all cases, is of viral origin [4,20]. That is why the assumption that it must be mostly the antiviral resistance enhancement which makes colostrum effective in preventing these infections seems to be valid. Another important statement is that natural immunity plays a much lesser role in viral infections than the adaptive one. Although the URTI is inevitably leading to an adaptive immunity reaction in the form of inflammation, this circumstance is regarded as leading to the symptoms’ development rather than the removal of the attacking viruses [1,4,38,39]. Some role is usually attributed to interferon in decreasing the speed of virus replication, as it is an important component of natural response; however, such a slowdown of the infection development rate merely gives the necessary time for a specific immune response to develop [38]. Therefore, according to the current knowledge, the adaptive immune response seems to be a leading factor in antiviral defense [38,40].

There are two types of adaptive immune responses involved in antiviral reactions: the usually initiating antiviral adaptive response is cellular cytotoxicity based on the action of CD8+ T cells, and the humoral response depends on the cooperation of CD4+ T-cells and B cells. The antibodies produced by plasma cells (the most mature form of B cells) against viral antigens are the most abundant and are therefore a highly effective tool for neutralizing viruses in both antiviral prevention and terminating the active viral infections. Regarding the above, it can be assumed that the effective prevention of URTIs due to colostrum supplementation should be attributed to adaptive immunity, which has been partly confirmed by the literature reporting on the effectiveness of colostrum and its individual components on the modulation of the adaptive immune system [41].

The antiviral action of adaptive immunity requires prior immunization against antigens of the particular pathogen. Such a process can happen via several modes of action: (1) immune reaction during active infection with some novel virus, (2) natural contact with viral antigens available in the environment in non-infectious form and (3) medicinal vaccination with viral antigens. The effective protection by the immune system (especially antibodies) can be obtained usually between 5 and 7 days from the first immunogenic contact with the virus. This time is required to develop the primary immune response, and before the process is complete, the infection develops with little obstacles, and its course depends mostly on the general fitness of the patient and relatively limited actions of natural immunity. What can potentially be improved by colostrum supplementation in such a case is the ability of the immune system to produce a primary immune response as quickly as possible. Various components of colostrum have been found to positively modify the development and efficiency of adaptive immunity [34,42].

The time required for the immune-response development can be largely decreased when the patient has immunogenic contact with a particular viral antigen prior to contracting it in infectious form. In such a case, the effective response may develop even within 2–3 days and is called a secondary immune response. This is obtained either over the course of a previous infection with a particular virus or alternatively due to natural, as well as medicinal, vaccination, and it may lead to a shorter and milder course of disease. Natural vaccination is most commonly a case of ingesting the inactivated viral antigens, followed by an immunization process happening within the intestinal immune system, which is based mostly on Peyer’s patches [43]. This process is highly efficient and very accurate as long as the intestinal homeostasis is preserved. Events that disrupt intestinal health, such as increased permeability of the intestinal barrier and dysbiosis, can lead to local inflammation and impair the steady and precisely regulated process of immunization in the gut, which is mediated by cytokines.

The highest level of protection from adaptive immunity, which practically eliminates the possibility of developing the disease, is achieved when the concentration of specific antiviral immunoglobulins in the blood serum and other body fluids is high enough. Such a protection from viral infection usually lasts for several months to a year from the last effective immunization. This implies that in order to remain free of the broad range of infections, regular contact with stimulating antigens of a wide variety needs to be maintained continuously. Because this contact occurs mostly with ingested antigens, it is necessary to constantly maintain intestinal homeostasis, in addition to ensuring the overall efficiency of the immune system in general terms.

Colostrum has been repeatedly proven to be among the most efficient products capable of restoring and maintaining intestinal homeostasis [44,45]. COL is known to have high healing potential, probably due to its high EGF (epidermal growth factor) and VEGF (vascular endothelial growth factor) content. In addition, some colostrum components, such as lactoferrin, proline-rich polypeptide, cytokines, growth factors and others, are known to modulate the immune system, making its reactions better regulated and more effective [35]. This may be of high importance, not only when antigenic stimulation in the gut is happening over the course of natural immunization. Enhancing the efficiency of anti-infectious immunity is also crucial during active infection to ensure that both primary and secondary immune responses are quick and effective. Any delay in counteracting the viral infections may lead to an unnecessary risk of various complications, with the secondary bacterial infections being among the most prominent.

Supplementation with colostrum may have one more beneficial effect, which is a positive influence on the general fitness. This is obtained partly through improving the function of the digestive tract, which allows for better nutritional status. Furthermore, although colostrum itself is of limited nutritional value, especially in small supplementation doses, it contains all extrinsic amino acids, as well as a relatively high concentration of several vitamins, minerals and other microelements. This may help to replenish the body’s resources. Several of those supplementary elements are also known to help improve the immune system’s status [42].

### 4.4. There Were No Side Effects from Colostrum Use

One of the most important concerns of investigators when introducing a novel medical procedure in children is the potential of triggering unexpected results. In the past, the use of colostrum has been blamed for causing side effects such as gastrointestinal symptoms, including abdominal pain, bloating and diarrhea. The symptoms usually last for the first few days of supplementation and are most probably related to the repair processes in the gut, as such processes naturally involves some acute inflammatory events. Therefore, we urged parents and guardians to pay special attention to any unusual symptoms appearing in their children. Over the entire trial period, none of such effects was reported. In addition, all parents and guardians responded to the question pointing specifically to abdominal symptoms. There was no statistically significant difference between the COL group and the PBO group in this regard (Figure 4).

### 4.5. Strengths and Limitations

The strengths of our study include the following: (1) RCT design with triple blinding, (2) short duration of supplementation period critical to boost immunity and (3) continuous outcome reporting. The limitation is a relatively limited sample size.

## 5. Conclusions

Our study provides the first solid RCT-based evidence that bovine colostrum can be effectively used to prevent and decrease the gravity of URTIs in pre-school children. The results we obtained allow us to conclude the following:

1. Kindergarten children 4–7 years of age receiving the pre-season colostrum supplementation may have up to 31% less days with URTI self-reported symptoms over 20 weeks from beginning of supplementation than children receiving a placebo.
2. These children tested within the same period (20 weeks) presented an even bigger median reduction in their URTI gravity score (−37%) in the COL vs. the PBO groups, which supposedly results not exclusively from sick days’ reduction but also from milder course of the URTIs.
3. The number of episodes, defined as 3 consecutive days of second degree of URTI gravity separated from other episodes by at least 3 days, was reduced in the COL group by 50% as compared to the PBO group over the entire period of the trial (21 weeks).
4. The observed effects of our study were obtained with main supplementation lasting for less than just 1/3 of the trial period.
5. There were no significant side effects observed during the trial, neither in the COL nor in the PBO groups. Moreover, there was no statistically significant difference between these groups in regard to gastrointestinal symptoms.

Overall, it can be concluded that our typical mild (15 days 2 × 500 mg + 30 days 1 × 500 mg) pre-seasonal supplementation with bovine colostrum provides significant protection to pre-school children from both frequency and gravity of URTIs over 140 days (20 weeks) of the fall/winter infectious season. The outcome of our study also indicates that the use of bovine colostrum in order to protect pre-school children from URTIs may have very important socioeconomic influence on the lives of their relatives and caregivers.

## Figures and Tables

**Table 1:** Description of the major outcomes of the study.

The Outcome	Acronym	Method of Calculation	Assessed at Weeks
Frequency of days with URTI symptoms	FSD	Number of days with any URTI symptoms/days of analyzed period	4, 8, 12, 14, 16, 18, 20, 21
Average gravity score of URTI symptoms	AGS	Total URTI score/days of analyzed period	4, 8, 12, 14, 16, 18, 20, 21
Number of URTI episodes	NUE	Sequence of days with URTI score 2 lasting for at least 3 days with at least 3 days separation from other episodes	21

**Table 2:** Baseline characteristics of analyzed children.

Variable	Colostrum			Placebo			*p*
	*N*	Median	IQR	*n*	Median	IQR	
BMI (kg/m^2^)	35	14.88	14.01–15.69	22	15.32	14.18–16.02	0.25
Age (years)	35	4	4.00–5.00	22	4	3.00–5.00	0.92
Mass (kg)	35	17	16.00–19.00	22	17	15.00–20.00	0.91

**Table 3:** The summary median reduction in the frequency of days with any URTI symptoms in the COL group versus the PBO group over various periods of the experiment.

Variable	COL		PBO		* p *	Median FSD Change COL vs. PBO
	Median	25–75 P	Median	25–75 P		
FSD 4 weeks	0.50	0.232–0.812	0.75	0.536–0.893	0.092	−33%
FSD 8 weeks	0.45	0.286–0.714	0.63	0.464–0.839	0.036	−30%
FSD 12 weeks	0.44	0.318–0.658	0.66	0.429–0.821	0.047	−33%
FSD 14 weeks	0.45	0.304–0.663	0.68	0.439–0.827	0.050	−34%
FSD 16 weeks	0.42	0.288–0.643	0.68	0.411–0.759	0.025	−37%
FSD 18 weeks	0.41	0.258–0.603	0.65	0.413–0.722	0.027	−37%
FSD 20 weeks	0.41	0.232–0.598	0.59	0.386–0.693	0.047	−31%
FSD 21 weeks	0.43	0.221–0.588	0.58	0.367–0.673	0.052	−26%

**Table 4:** The summary AGS medians’ reduction in the COL group versus the PBO group over various periods of experiment.

Variable	COL		PBO		* p *	Median AGS Change COL vs. PBO
	Median	25–75 P	Median	25–75 P		
AGS 4 weeks	0.50	0.259–0.884	0.91	0.643–1.071	0.016	−45%
AGS 8 weeks	0.50	0.304–0.777	0.79	0.500–1.018	0.024	−36%
AGS 12 weeks	0.51	0.396–0.801	0.77	0.488–1.000	0.036	−33%
AGS 14 weeks	0.49	0.362–0.786	0.82	0.561–1.020	0.027	−40%
AGS 16 weeks	0.48	0.335–0.732	0.78	0.500–0.982	0.019	−38%
AGS 18 weeks	0.48	0.310–0.720	0.80	0.492–0.960	0.026	−41%
AGS 20 weeks	0.47	0.279–0.716	0.75	0.493–0.893	0.041	−37%
AGS 21 weeks	0.46	0.265–0.709	0.73	0.483–0.864	0.052	−36%

## Data Availability

Data are available upon request.
